# Evaluation of the Analgesic Efficacy of Preemptive Dipyrone in the Postoperative Period of Adenotonsillectomy in Children: A Randomized Clinical Trial

**DOI:** 10.1055/s-0046-1827208

**Published:** 2026-10-14

**Authors:** Maira I. S. Stangler, José F. Lubianca Neto, Marcelo N. Lubianca

**Affiliations:** 1 Graduate Program of Pediatrics at UFCSPA, Federal University of Health Sciences of Porto Alegre (UFCSPA), Porto Alegre, RS, Brazil; 2 Medical School of Federal University of Health Sciences of Porto Alegre (UFCSPA), Graduate Program of Pediatrics at UFCSPA, Pediatric Otorhinolaryngology Service at Santo Antônio Children's Hospital, Porto Alegre, RS, Brazil; 3 Medical School of Pontifical Catholic University of Rio Grande do Sul, Porto Alegre, RS, Brazil

**Keywords:** adenotonsillectomy, child, surgery, pain, dipyrone

## Abstract

**Introduction:**

Dipyrone is one of the most commonly used postoperative analgesics. However, there are only two randomized, placebo-controlled clinical trials evaluating its effect in pediatric tonsillectomies.

**Objective:**

To compare postoperative pain outcomes following the use of pre-incisional dipyrone or placebo in pediatric adenotonsillectomy.

**Methods:**

This was a randomized, double-blind, parallel, placebo-controlled clinical trial in children aged 4 to 14 years who underwent adenotonsillectomy. A total of 76 participants were randomized into the intervention/dipyrone group (n = 39) and the placebo group (n = 37). All received either dipyrone or saline solution (placebo) at one of two time points: before the surgical incision or at the end of surgery.

**Results:**

The mean age was 6.8 years and weight was 32.1 kg. In the intervention group, vital signs were normal and only 7.7% experienced emesis, compared with 8.1% in the placebo group. Food intake was anticipated by 1,1 hours in the dipyrone group. Pain was controlled in at least 77.6% of participants at all evaluated time points (H0, H1, H3, and H6) in both groups. At H3, pain was controlled in 54.2% of the intervention group compared with 45.8% in the placebo group. In addition, the dipyrone group demonstrated a tendency toward a lower need for opioids in H1 and H3, reducing opioid administration by 50% and 52.63%, respectively, compared with placebo.

**Conclusion:**

A trend toward pain relief, earlier diet acceptance, and a lower need for opioids was observed in the group that received preemptive dipyrone. The results did not reach statistical significance, likely due to a type II error.

## Introduction

Tonsillectomy, with or without adenoidectomy, is one of the most frequent surgeries in pediatric practice. 1 2 It is considered a safe procedure, and patients are typically discharged approximately six hours after the intervention. Several techniques can be used, each with its advantages and disadvantages. 1 3 The indications for tonsillectomy are divided into absolute and relative. Among the absolute indications are tonsillar hyperplasia, obstructive sleep apnea syndrome (OSAS), and suspected malignancy. Chronic respiratory obstruction resulting from adenotonsillar hyperplasia may lead to language alterations, feeding difficulties, dental malocclusion, and changes in orofacial growth. When associated with OSAS, obstruction may also cause poor-quality sleep, agitation, and snoring, potentially leading to growth delay, cognitive deficits, mood changes, pulmonary hypertension, and other complications. Unlike in adults, treatment of OSAS in children is curative in most cases, since the main cause is hyperplasia of the adenoids and/or palatine tonsils. However, postoperative follow-up is essential, as recurrence of obstruction may occur and a porpotion of cases do not achieve resolution of OSAS. 4 There are also relative indications for tonsillectomy, such as recurrent tonsillitis, tonsillitis associated with systemic diseases, peritonsillar abscess, and halitosis due to caseous tonsillitis. 5

The most common complications of tonsillectomy are nausea, vomiting, bleeding, and postoperative pain. Such complications may increase hospital stay and the need for medication. The incidence of delirium and/or agitation upon emergence from anesthesia is high. 6 These may be caused by rapid return of consciousness in an unfamiliar environment, presence of pain, stress during induction, hypoxemia, airway obstruction, noisy environment, duration of anesthesia, child temperament, use of pre-anesthetic medication, and the anesthetic technique used. 7 These factors may cause stressful and negative behavioral changes in children. 8

Controlling acute postoperative pain is a priority, as it directly affects the child's recovery and impacts basic needs such as sleep, rest, feeding, and interaction. 9 Studies are needed to evaluate the effect of analgesic pharmacological interventions applied at different intraoperative stages on postoperative clinical outcomes, assessing their impact on the quality of postoperative recovery after tonsillectomy.

Although dipyrone is the most widely used postoperative analgesic in children in countries where its use is approved, few studies quantitatively assess its effect. There are also few studies using appropriate methodology. In the case of adenotonsillectomy in children, high-quality evidence for the use of dipyrone is even scarcer. Only two randomized, placebo-controlled clinical trials evaluate the analgesic effect of this medication in pediatric adenotonsillectomy: one assessing the preemptive analgesic effect 10 and the other evaluating postoperative administration. 11 The only study assessing the preemptive effect of dipyrone demonstrated efficacy in pain relief during the first 6 postoperative hours and lower cumulative need for pethidine. The present study aimed to evaluate postoperative pain relief associated with preemptive dipyrone in children undergoing adenotonsillectomy. Notably, this is the second clinical trial to evaluate dipyrone as preemptive analgesia in tonsillectomy and the only one studying dipyrone versus placebo exclusively in the pediatric population.

## Objective

To compare postoperative pain outcomes following pre-incisional administration of dipyrone or placebo in pediatric adenotonsillectomy.

## Methods

A randomized, double-blind, parallel, placebo-controlled clinical trial was conducted in the surgical center of Hospital da Criança Santo Antônio, in Porto Alegre, Brazil, approved by the Ethics Committee of **Hospital da Criança Santo Antônio** on October 31, 2018, under Opinion No. 2.993.615 and CAAE: 98245418.2.0000.5683.

The surgical center is a reference for the pediatric population treated by the Brazilian Unified Health System (SUS) in Porto Alegre. Sample size was obtained using a free tool available at https://comentto.com/calculadora-amostral/ . A type I error of 5% and a statistical power of 90% were considered. The final calculated sample was 121 patients. The study period covered the years 2018 to 2021. However, in 2019 and 2020, due to the COVID-19 pandemic, elective surgeries—including adenotonsillectomy—were suspended. Consequently, no additional patients could be included. In 2021, surgical activity resumed minimally, leading to the closure of the study due to the impossibility of further patient inclusion.

To select the patients who would compose the database, an initial clinical evaluation of the scheduled children was performed upon arrival at the surgical center. This evaluation included verification of fasting time, clinical conditions (such as absence of fever, pain, or other pathologies preventing surgery), authorization from guardians, and appropriate age. Only after this screening were eligible patients included in the database as the study population. The total sample comprised 76 children aged 4 to 14 years, of both sexes, with indication for tonsillectomy with or without adenoidectomy, whose guardians (aged over 18 years) were present at the time of consent (Informed Consent Form [ICF] and Assent Form [AF]). The 76 participants were randomized in blocks of 6 individuals to maintain a similar number of patients in each group (39 children in the intervention group and 37 in the placebo group). The pharmacist created 13 groups (letters A to M), with randomization performed using the Random Allocation software, version 1.0 (Isfahan, Iran). On the day of the procedure, the interventionist (anesthesiologist) received a 2-mL syringe labeled with code No. 1 (to be administered during induction) or No. 2 (to be administered at the end of surgery). It is important to note that syringe No. 1 indicated preemptive analgesia, which must be administered before any surgical injury or incision. After the child entered the surgical center and guardian authorization was obtained, the pharmacist prepared the syringes according to the pre-generated scheme, using the randomized drug (dipyrone) and/or placebo (saline solution), with possible variation between syringes No. 1 and No. 2. Importantly, upon awakening, all children received morphine as analgesia, regardless of randomization, to avoid harming those who received placebo in this phase. Blinding was maintained throughout all phases of the study among the researchers, anesthesiologists, and surgeons.

Meanwhile, in the preoperative room, parents completed a demographic questionnaire, the sleep scale (SBQ), 12 the pain scale (VAS), and received postoperative instructions in the recovery room. At the end of surgery, the child was taken to the recovery room, where a previously trained nursing technician assessed pain using validated scales: the CHIPPS Scale (Children and Infants Postoperative Pain Scale) 13 for children aged 0 to 5 years, and the Visual Analog Scale (VAS) 14 for older children aged 6 to 14 years, at the following time points: H0 (arrival), H1 (1 hour after surgery), H3 (3 hours after surgery), and H6 (6 hours after surgery). In the postoperative period, pain was classified as mild (not requiring dipyrone rescue) if scores ranged from 1 to 3. Opioid rescue was indicated for scores above 7. To avoid measurement bias, the pharmacist, anesthesiologists, and nursing technicians were trained by the principal investigator.

Inclusion criteria were ages 4 to 14 years, ASA physical status I (healthy patient) or II (patient with mild disease); first scheduled tonsillectomy with or without adenoidectomy; signed informed consent from the guardian and assent when applicable. Exclusion criteria were previous adenoidectomy; any chronic disease requiring fixed daily analgesia; developmental delay; inability to understand Portuguese; absence of cohabiting parents; and refusal to participate.

Descriptive analysis was performed to characterize the sample. Qualitative variables were presented as absolute and relative frequencies, and quantitative variables as mean and standard deviation (SD). To compare intervention and placebo groups, Chi-square or Fisher's exact test and Student's *t* -test were used, depending on the variable. All analyses were performed using R software, version 4.1.3 (R Foundation for Statistical Computing, Vienna, Austria). A p-value of < 0.05 was considered statistically significant.

## Results

Fig. 1 presents the study flowchart. There were no participant losses or missing data. This was facilitated by the fact that all study data was obtained during hospitalization, before the patient's discharge, either in the operating room or the recovery room.

**Fig. 1:**
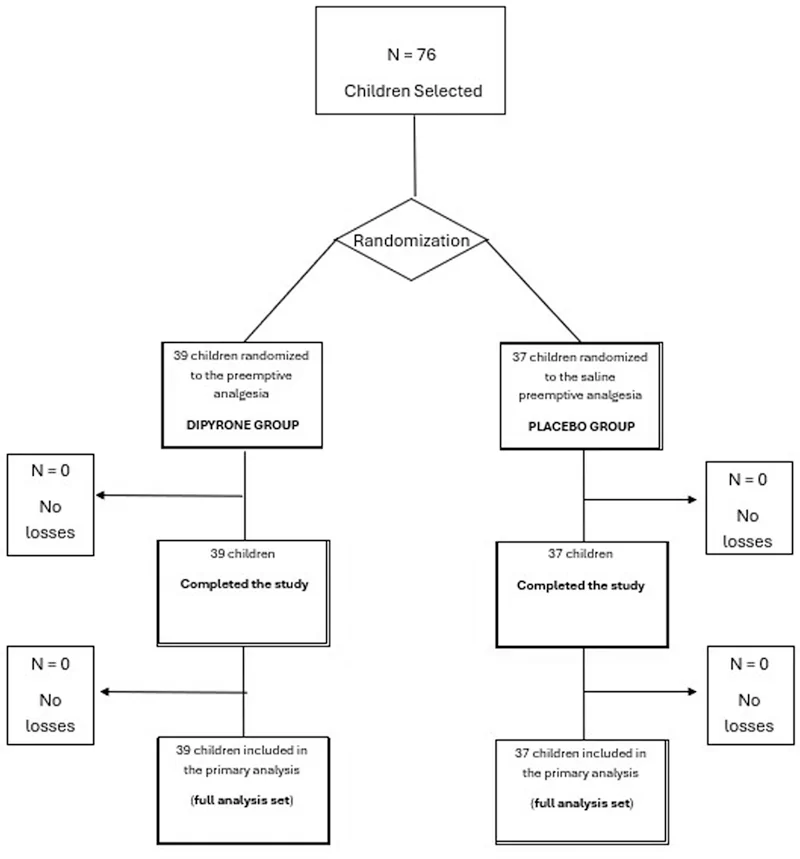
Flow of participants in the study.

The sociodemographic characteristics of the sample according to randomization group are presented in Table 1 . Mean age was 6.8 years (SD = 2.7) and mean weight was 31.2 kg (SD = 14.0). Most individuals (92.0%) attended school, and there was no statistically significant difference in educational level between groups. The placebo group showed higher school attendance, and their parents more frequently completed high school compared with the preemptive dipyrone group (87.2% versus 97.3% and 41.0% versus 75.7%, respectively). Other characteristics such as diseases, previous hospitalizations and surgeries, and use of continuous medication were similarly distributed between groups. Regarding comorbidities, 56.6% of patients had previous diseases, the most frequent being rhinitis (41.9%) and asthma (34.9%). Additionally, 28.9% of the sample used continuous medication, mainly intranasal budesonide and fluticasone, each used by 22.7% of patients.

**Table 1:** Sociodemographic characteristics of participants according to randomization group

Characteristics of the sample*	Total ( *n* = 76) n (%)	Dipyrone ( *n* = 39) n (%)	Placebo ( *n* = 37) n (%)
**Age** a	6.8 (± 2.7)	6.4 (± 2,3)	7.2 (± 3,0)
**Weight** a	31.2 (± 14.0)	30.1 (± 14.2)	32.4 (± 13.9)
**Sex**			
Female	42 (55.3)	24 (61.5)	18 (48.6)
Male	34 (44.7)	15 (38.5)	19 (51.4)
**School attendance rate**			
No	6 (7.9)	5 (12.8)	1 (2.7)
Yes	70 (92.1)	34 (87.2)	36 (97.3)
**ASA**			
1	69 (90,8)	34 (87.2)	35 (94.6)
2	7 (9.3)	5 (12.8)	2 (5.4)
**Previous disease**			
No	33 (43.4)	18 (46.2)	15 (40.5)
Yes	43 (56.6)	21 (53.8)	22 (59.5)
**Previous hospitalization**			
No	42 (55.3)	22 (56.4)	20 (54.1)
Yes	34 (44.7)	17 (43.6)	17 (45.9)
**Previous surgery**			
No	60 (79.0)	31 (79.5)	29 (78.4)
Yes	16 (21.1)	8 (20.5)	8 (21.6)
**Use of continuous medication**			
No	54 (71.1)	28 (71.7)	26 (70.2)
Yes	22 (28.9)	11 (28.2)	11 (29.7)
**Caregiver**			
Mother	65 (85.5)	33 (84.6)	32 (86.5)
Father	11 (14.5)	6 (15.4)	5 (13.5)
**Caregiver's educational level**			
Elementary/middle/high school	32 (42.1)	23 (59.0)	9 (24.3)
Undergraduate/Technical/Specialization	44 (57.9)	16 (41.0)	28 (75.7)

*There was no statistically significant difference in the distribution of characteristics between groups.

a mean (standard deviation ).

Table 2 describes vital sign measurements. Overall, measurements were similar between intervention and placebo groups, demonstrating baseline balance.

**Table 2:** Children's vital signs

Characteristics a	Total ( *n* = 76)	Intervention ( *n* = 39)	Placebo ( *n* = 37)
**Axillary Temperature**	36.3 (0.2)	36.3 (0.3)	36.3 (0.2)
**Respiratory Rate**	21.8 (2.5)	22.0 (2.3)	21.5 (2.7)
**Heart Rate**	96.6 (16.4)	98.6 (14.7)	94.5 (18.1)
**Hemoglobin Oxygen Saturation**	98.0 (1.5)	98.2 (1.5)	97.8 (1.6)

a mean (standard deviation).

Postoperative symptoms and time to diet acceptance are described in Table 3 . Nausea occurred in 9.2% of cases, vomiting in 7.9%, and fever in 5.3%. There was no difference in incidence between groups. Mean time to diet acceptance was 2.9 hours (SD = 3.4). In the placebo group, time to accept diet was longer than in the intervention group (3.5 versus 2.4 hours), but this difference was not statistically significant.

**Table 3:** Assessment of postoperative symptoms

Characteristics	Total ( *n* = 76)	Intervention ( *n* = 39)	Placebo ( *n* = 37)	*p* -value
	n (%)	n (%)	n (%)	
**Nausea**				0.9998
Yes	7 (9.2)	4 (10.3)	3 (8.1)	
No	71 (90.8)	35 (89.7)	34 (91.9)	
**Vomiting**				0.9998
Yes	6 (7.9)	3 (7.7)	3 (8.1)	
No	70 (92.1)	36 (92.3)	34 (91.9)	
**Fever**				0.1156
Yes	4 (5.3)	4 (10.3)	0 (0.0)	
No	72 (94.7)	35 (89.7)	37 (100.0)	
** Diet acceptance (in hours) a**	2.9 (3.4)	2.4 (1.4)	3.5 (4.6)	0.1575

a mean (standard deviation).

Pain and sleep assessment results are shown in Table 4 . There was no significant difference between groups regarding pain or sleep, and pain remained constant, with at least 77.6% of children reporting pain at all time points. Comparing groups, pain control was achieved in 54.2% of the intervention group and 45.8% of the placebo group, with the greatest difference at H3. According to parents' assessment of pain/discomfort during sleep, 64.5% reported unsatisfactory sleep, consistent with the SBQ questionnaire, which showed only 14.5% rating sleep as “GOOD,” compared with 87.17% “FAIR” in the dipyrone group and 78.37% in the placebo group.

**Table 4:** Assessment of pain and sleep using the CHIPPS Scales; VAS and SBQ Questionnaire

Scales	Total ( *n* = 76)	Intervention ( *n* = 39)	Placebo ( *n* = 37)	*p* -value
	n (%)	n (%)	n (%)	
**CHIPPS or VAS depending on age.**				
**H0 (on arrival)**				0.998
Controlled pain	69 (90.8)	35 (50.7)	34 (49.3)	
Need treatment	7 (9.2)	4 (57.1)	3 (42.9)	
**H1 (1 hour after arrival)**				0.370
Controlled pain	63 (82.9)	34 (54.0)	29 (46.0)	
Need treatment	13 (17.1)	5 (38.5)	8 (61.5)	
**H3 (3 hours after arrival)**				0.415
Controlled pain	59 (77.6)	32 (54.2)	27 (45.8)	
Need treatment	17 (22.4)	7 (41.2)	10 (58.8)	
**H6 (6 hours** **after arrival)**				0.998
Controlled pain	71 (93.4)	36 (50.7)	35 (49.3)	
Need treatment	5 (6.6)	3 (60.0)	2 (40.0)	
**Child's sleep quality as assessed by** **parents using the VAS**				0.161
Unsatisfactory	31 (40.8)	20 (64.5)	11 (35.5)	
Moderate	24 (31.6)	10 (41.7)	14 (58.3)	
Satisfactory	21 (27.6)	9 (42.9)	12 (57.1)	
**Sleep Scale (SBQ)**				0.665
Good	11 (14.5)	4 (36.4)	7 (63.6)	
Regular	63 (82.9)	34 (54.0)	29 (46.0)	
Terrible	2 (2.6)	1 (50.0)	1 (50.0)	

Fig. 2 shows opioid use at different time points (H0, H1, and H3) for both groups. An increase in opioid use was observed over time in the placebo group, while it remained stable and low in the dipyrone group.

**Fig. 2:**
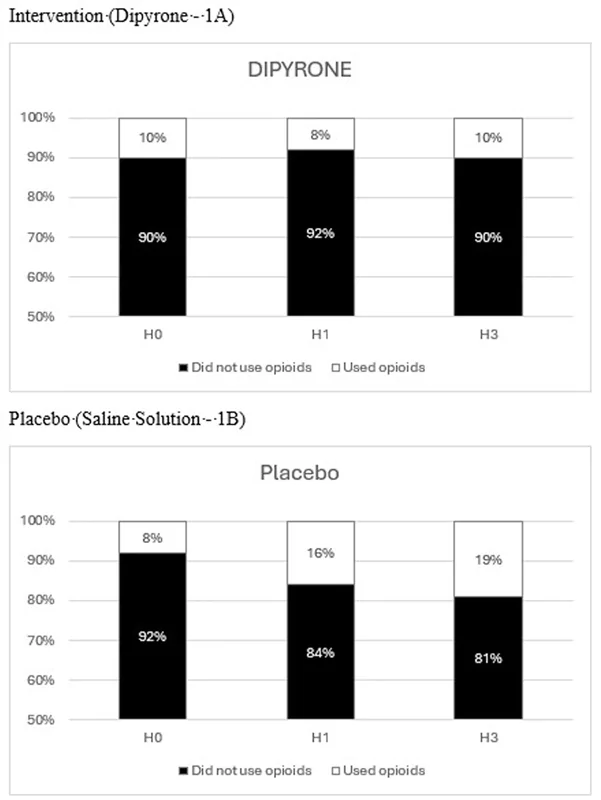
Opioid use at time points H0, H1, and H3 in the intervention (dipyrone; panel A) and placebo (saline solution; panel B) groups.

One of the strengths of this study was the choice of research design. The randomized clinical trial is the study with the greatest ability to discriminate between interventions, as most systematic and random biases can be controlled during the planning phase. Randomization enables unbiased allocation by preventing researcher involvement. Analysis of sociodemographic variables and vital signs indicates that randomization produced comparable groups. The only two randomized clinical trials evaluating dipyrone for postoperative tonsillectomy pain 10 11 included children aged 3 to 15 years, similar to our sample (4 to 14 years). Mean heart rate (96.6 beats/min) and respiratory rate (21.8 breaths/min) were similar to population standards from the Brazilian Ministry of Health's Child Health Handbook, 15 supporting generalizability.

As in Kocum 2013, 10 nausea and vomiting incidence was similar between groups and low (< 10%). These factors did not increase time to food intake. Although the intervention group accepted diet about one hour earlier than the placebo group (2.4 versus 3.5 hours), the difference was not statistically significant.

Pain was controlled in more participants in the intervention group at all time points (H0, H1, H3, H6) ( Table 4 ), although without statistical significance. This trend aligns with the findings of Kocum et al. 10 and Sener et al. 11 The former showed greater pain relief in the dipyrone group during the first 6 postoperative hours, while the latter confirmed significantly lower VAS scores in the intervention group. It is important to note that in Kocum (2013), two concomitant analgesics were used before surgery. In the present study, the analgesic effect can be considered specific to dipyrone. Although the absence of combined medications may partially explain the lack of statistical significance, the most likely explanation is the low statistical power due to premature study interruption caused by the COVID-19 pandemic.

Regarding opioid use, Fig. 2 shows a significant reduction in the intervention group during the first postoperative hour. Sener et al. 11 also found this difference, and Kocum et al. 10 found lower opioid use in the intervention group, although in both studies the effect appeared only at the sixth postoperative hour. In the present study, preemptive dipyrone had a more notable effect at the first and third postoperative hours, reducing opioid administration by 50% and 52.63%, respectively, compared with placebo. This clear trend, however, did not reach statistical significance, likely due to type II error.

## Conclusion

Although statistically significant differences were not found, most results indicate a trend toward better pain relief with preemptive dipyrone. Better pain control was observed (as evidenced by VAS scores), along with a reduced need for rescue opioids and earlier diet acceptance by approximately one hour in the dipyrone group. The incidence of adverse effects such as nausea and vomiting was low and similar in both groups. This is only the second clinical trial in the literature evaluating the effect of dipyrone as preemptive analgesia for tonsillectomy. Considering that dipyrone is an accessible, low-cost analgesic with few adverse effects and widely used in Brazil, more randomized placebo-controlled clinical trials in the pediatric population are needed.
